# Anthropometric indicators as a discriminator of sarcopenia in community-dwelling older adults of the Amazon region: a cross-sectional study

**DOI:** 10.1186/s12877-020-01923-y

**Published:** 2020-12-01

**Authors:** Cássio Lima Esteves, Daniela Gonçalves Ohara, Areolino Pena Matos, Vânia T. K. Ferreira, Natalia C. R. Iosimuta, Maycon Sousa Pegorari

**Affiliations:** Physical Therapy Course, Department of Biological and Health Sciences, Federal University of Amapá, Road Juscelino Kubitschek, Km – 02, Jardim Marco Zero, Macapá, Amapá CEP 68903-419 Brazil

**Keywords:** Sarcopenia, Anthropometry, Older adult health, Urban population

## Abstract

**Background:**

Sarcopenia is a geriatric syndrome associated with negative health outcomes and the use of viable alternative screening tools may help in the diagnosis of this condition. This study aimed to analyze the association of sarcopenia with anthropometric indicators among community-dwelling older adults and to identify cut-off points for such indicators as a discriminant criterion for predicting sarcopenia.

**Methods:**

This was a cross-sectional study conducted on community-dwelling older adults ≥60 years old (*n* = 411) of both sexes from Macapá, Amapá, Brazil. Socioeconomic, clinical and anthropometric data (arm circumference - AC, waist circumference - WC, calf circumference - CC and body mass index – BMI) were collected using a structured form. Sarcopenia was identified according to the EWGSOP 2 consensus. The association between anthropometric indicators and sarcopenia was performed using logistic regression and cut-off points established from the ROC Curve. Statistical significance was defined as *p* ≤ 0.05.

**Results:**

Adjusted analysis indicated an independent and inverse association between sarcopenia and the anthropometric indicators: AC (odds ratio, OR: 0.63; 95% confidence interval, 95%CI: 0.53–0.76), CC (OR: 0.73; 95%CI: 0.62–0.85), WC (OR: 0.93; 95%CI: 0.90–0.97) and BMI (OR: 0.64; 95%CI: 0.53–0.76). The following cut-off points for older men and women represented the discriminant criterion for the presence of sarcopenia: WC (≤97 and ≤ 86 cm), CC (≤33 and ≤ 31 cm), AC (≤27 cm) and BMI (≤24.8 kg/m^2^ and ≤ 24.5 kg/m^2^) (area under the ROC curve superior to 0.70). BMI and AC were the indicators with the highest ability to discriminate older adults of both sexes with sarcopenia.

**Conclusions:**

An increase of one unit of the indicators can reduce the probability of occurrence of sarcopenia. All indicators were considered to discriminate the occurrence of sarcopenia, with emphasis on BMI and AC, and could be used to screen for this condition among community-dwelling older adults.

**Supplementary Information:**

The online version contains supplementary material available at 10.1186/s12877-020-01923-y.

## Background

Sarcopenia is a muscle disease with cumulative characteristics in lifetime, defined by low levels of muscle strength, muscle quantity/quality and physical performance [1]. The condition is associated with negative health outcomes among older adults, impairing functional ability and the quality of life, causing falls and fractures, and involving high health care costs and mortality rates [2]. Its prevalence is 1 to 29% among community-dwelling older adults [2] and about 17% in the older Brazilian population [3].

In view of the impact of sarcopenia on the public health area, an early identification of the condition becomes relevant [4]. The diagnosis can be made by determining a reduction of muscle mass accompanied by reduced muscle strength and/or reduced physical performance, the latter conditions being assessed by dynamometry and gait speed, respectively [5]. Dual-energy X-ray emission absorptiometry (DXA), magnetic resonance (MR) or computed tomography (CT) are specifically recommended for the assessment of low muscle quality and quantity. However, these are expensive methods involving the risk of exposure to radiation and are little available to the public [6]. Thus, simpler low-cost, noninvasive and easily applicable methods such as anthropometry may represent viable alternative screening tools helping the diagnosis of sarcopenia [7, 8].

Several studies have pointed out the use of anthropometry for the screening of sarcopenia [5, 8–10], however these studies has been performed in different countries with different life-style as Australia, Korea, Japan and Turkey, which make difficult generalize data from them to use in South American countries. In addition, the World Health Organization (WHO) [11] considers calf circumference (CC) to be a more sensitive anthropometric index of muscle mass among older adults, although arm circumference (AC), Abdominal circumference (AbC) and body mass index (BMI) have also been used and documented in the scientific literature as predictors of sarcopenia [9, 12].

Studies conducted in Brazil are recent and have determined the viability of CC as a discriminator of muscle mass in older adults [13, 14], as well as BMI [15], waist-height ratio and waist circumference identified with DXA [7]. Besides, the majority of this studies have been mostly performed and developed in specifics regions or states in Brazil [3]. Thus, there are few studies conducted in the northern region of Brazil that investigate how these older adults are getting older and if they are sarcopenic. This information is important, mainly because the Northern Brazil region, or more specifically in Macapá city, the Amazon region, is located in one of the least developed regions of the country, which fortify the technological resource scarcity, considered the gold standard instrument to assess sarcopenia.

According to data estimated [16], in the year 2010, the city of Macapá had 16% of the population living in the subnormal agglomerations, or places with no planning and situated in areas considered as inappropriate called areas of “ressaca”, “baixadas” or stilt. The areas of “ressaca” cover 20% of the city’s urban perimeter [17], “they behave like natural water reservoirs in a complex and distinct ecosystem and suffering the effects of tidal action through an intricate network of canals and streams plus the seasonal rain cycle” [18]. For this, the subnormal agglomerations areas are locations which are low socioeconomic conditions and difficult to access readily, which make hard the dislocation of the residents from their houses to hospitals or the access of basic healthcare professionals. Thus, there is an urgent necessity to adopt easy-to-use instruments as anthropometric indicators to identify geriatrics syndromes as sarcopenia in these areas, once it is impossible take huge or expensive equipment in these areas.

In view of the scarcity of studies validating anthropometric measures as screening tools for sarcopenia among older persons, according to Cruz-Jentoft et al. [19], it is to be believed that such indicators may represent a viable and additional alternative to be used to facilitate screening in order to guide the diagnosis of sarcopenia and the appropriate interventions, with an impact on health care for the older population, especially in areas of the Brazilian Amazon region. Besides, validating the anthropometric measures and finding the cut-off points to determine the sarcopenia condition, it will be possible to analyze if this data is different from the other studies and understand the impact of environmental factors on the living conditions of population from the Northern Brazil region.

Thus, the objectives of the present study were to analyze the association between sarcopenia and anthropometric indicators among community-living older subjects and to identify cut-off points for the anthropometric indicators as a discriminant criterion for the prediction of sarcopenia.

## Methods

### Context and study population

This was a cross-sectional study conducted on 411 older adults residing in the urban area of Macapá, in 2017. Information about the characteristics of the population and sample calculation, as well as the procedures for data collection are available in a previously published study [20]. The present study was approved by the Research Ethics Committee (protocol n° 1.738.671).

The study was conducted on subjects aged 60 years or older who resided in the urban area of the municipality of Macapá, able to walk with or without help, and who gave written informed consent to participate. Subjects who could not be located after three attempts by the interviewer, who had moved to another city, who were hospitalized and who had neurological sequelae and/or conditions that would not permit their assessment, were excluded.

Also excluded were subjects with cognitive decline that would prevent them from responding to the questions of the interviewer and from performing the tests, as determined by the translated version of the Mini Mental State Examination (MMSE) validated for Brazil, which considers cut-off points based on schooling level [21].

### Instruments for data collection

#### Sarcopenia (dependent variable)

Sarcopenia was established using the operational definition recommended by the European Working Group on Sarcopenia in Older People (EWGSOP) 2 and the diagnosis considered the associated of low muscle strength and low muscle mass [19]:

1. Low muscle mass: The muscle mass component was measured based on the total muscle mass (TMM) estimated by the equation proposed by Lee et al. [22], validated for use in Brazilian elderly [23] and used in previous population-based studies [20, 24]: [MMT (kg) = (0.244 x body weight) + (7.8 x height) - (0.098 x age) + (6.6 x sex) + (ethnicity - 3.3)]. The equation considers the parameters body mass, height, sex, age and race. For the sex variable, 0 = women and 1 = men; for ethnicity, 0 = white and indigenous, − 1.2 = yellow and 1.4 = black and brown were adopted. Based on the TMM, the muscle mass index (MMI = TMM / height2) was calculated. The cut-off point for muscle mass index (MMI) in the present study considered the 20th percentile of the sample studied, according to previous studies [25, 26] and represented values < 9.61 kg/m2 for men and < 6.92 kg/m2 for women [20, 24].
2. Reduction of muscle strength was measured with a manual hydraulic dynamometer, SAEHAN® Hydraulic Hand Dynamometer, model SH5001, using handgrip strength (HGS) in an isometric manner based on kg/force (kgf) as recommended by the American Society of Hand Therapists [27]. Three measurements were made at one-minute intervals in the dominant limb and their mean value was considered. Values of less than 27 kgf for men and less than 16 kgf for women were considered to indicate reduced muscle strength [28].

#### Anthropometric indicators (independent variable)

The following anthropometric indicators were assessed in the present study: Body Mass Index (BMI), calf circumference (CC), waist circumference (WC), and arm circumference (AC). The perimeters/circumferences were measured with an inelastic tape. BMI was determined with a portable digital scale (Whole Body Control Scale, Omron, Model HBF 514C, 150 kg), with the subject barefoot and wearing the minimum amount of clothing possible. As recommended, the height was measured with the person barefoot, standing straight with joined feet and with the heels, buttocks and head in contact with the wall, keeping his eyes fixed on a horizontal axis parallel to the floor. BMI was defined as kg/height^2^ [29, 30].

AC was measured at the midpoint between acromion and olecranon in the arm of the subject resting against his body in a relaxed manner [31, 32]. CC can be measured on the point of largest perimeter of the right or left leg, with the subject sitting or lying in dorsal decubitus without contracting the calf muscles [33]. In the present study we measured the left leg with the subject in the sitting position.

WC was measured using the protocol recommended by the WHO [34], i.e., at the approximate midpoint between the lower margin of the last palpable rib and the top of the iliac crest. Other elements were considered like as posture, breathing phase and abdominal tension. The posture recommended here was orthostatic position, arms along the sides of the body, joined feet and weight uniformly distributed between them. WC should be measured at the end of a normal expiration, and abdominal tension at the measuring point should be avoided, i.e., the subject should remain relaxed during the measurement [34, 35].

#### Adjustment variables

Variables such as age, sex, schooling, income, health perception, number of diseases and medications, hospitalization, and the occurrence of falls in the last ear were recorded on a structured form. The subjects were asked to report the use of tobacco and its duration (years) (yes/no). Functional capacity was assessed using the Katz independence Scale for basic activities of daily life (BADL) [36] and the Lawton and Brody scale [37] for instrumental activities of daily life (IADL). Older adults who did not show difficulty in performing any BADL or IADL were considered to be independent, while subjects with difficulty in performing one or more activity were considered to be dependent. The level of physical activity was determined using the long version of the International Physical Activity Questionnaire (IPAQ) [33]. Subjects were considered to be sufficiently active when they engaged in vigorous physical activity for 150 min or more per week and subjects who engaged in 0 to 149 min of vigorous or moderate weekly activity per week were considered to be inactive. The complete version of the interview guide is available in Additional file 1.

### Statistical analysis

Data are reported as means, standard deviations, median (interquartile range), absolute number, and percentage. Comparative analysis between the sarcopenic and non-sarcopenic groups was carried out using the Student t-test and Mann Whitney U test, according to the data distribution verified by the Kolmogorov-Smirnov test, for the quantitative variables and the chi-square test for the categorical variables. The association between anthropometric indicators (independent variable) and sarcopenia (dependent variable) was determined by crude and adjusted analysis using the logistic regression model and the estimate of the odds ratio (OR), with the 95% confidence interval (95%CI), and the level of significance set at 5% (*p* < 0.05). The Hosmer and Lemeshow test (*p* > 0.05) was applied to analyze the degree of model fit. All analyses were carried out using the Statistical Package for the Social Sciences (SPSS) version 21.0.

Receiver Operating Characteristic (ROC) curves were constructed to determine the cut-off points of the anthropometric indicators as discriminators of sarcopenia, and the area under the ROC curve (AUC), the sensitivity and specificity were determined using the MedCalc 11.4.4 software, with 95%CI and a 5% level of significance (*p* < 0.05).

## Results

The final sample consisted of 411 older adults recruited on the basis of inclusion and exclusion criteria. The characteristics of the selected subjects are listed in Table 1. The prevalence of sarcopenia was 6.1% (*n* = 25). Sarcopenic subjects had lower values of anthropometric indicators than non-sarcopenic subjects (*p* < 0.05) (Table 1).

**Table 1:** Characteristics of the older adults according to sarcopenia

Variables	Sarcopenic (*n* = 25)	Non-sarcopenic (*n* = 386)	p* Value	Total sample (*n* = 411)
Age (years)	77.04 ± 8.99	69.69 ± 6.90	< 0.001	70.15 ± 7.25
Sex				
Male	10 (40)	128 (33.2)	0.483	138 (33.6)
Female	15 (60)	258 (66.8)		273 (66.4)
Height (m)	1.51 (1.46–1.58)	1.54 (1.48–1.60)	0.244	1.52 (1.48–1.60)
Weight (kg)	50.28 ± 7.96	67.86 ± 12.86	< 0.001	66.79 ± 12.29
Schooling (years)	3 (1.5–7)	5 (2–10)	0.202	4 (2–10)
Income				
None	1 (4)	43 (11.1)	0.311	44 (10.7)
1 minimum wage or less	15 (60)	178 (46.1)		193 (47)
2 minimum wages or more	9 (36)	165 (42.7)		174 (42.3)
Health perception				
Positive	8 (32)	116 (30.1)	0.844	124 (30.2)
Negative	17 (68)	269 (69.9)		286 (69.8)
MMI (kg/m^2^)	7.17 ± 1.56	9.02 ± 1.70	< 0.001	8.91 ± 1.74
HGS (kgf)	16.52 ± 4.73	25.18 ± 8.96	< 0.001	24.65 ± 9.01
Number of diseases	4 (3–7)	5 (3–7)	0.803	5 (3–7)
Number of medications	1 (0–2)	1 (0–3)	0.534	1 (0–3)
Falls in the last year				
Yes	2 (8)	81 (21)	0.117	83 (20.2)
No	23 (92)	305 (79)		328 (79.8)
Hospitalization in the last year				
Yes	5 (20)	53 (13.7)	0.485	58 (14.1)
No	20 (80)	333 (86.3)		353 (85.9)
Smoking habit				
Yes	4 (16)	35 (9.1)	0.289	39 (9.5)
No	21 (84)	351 (90.9)		372 (90.5)
Physical activity				
Sufficiently active	9 (36)	209 (54.1)	0.078	218 (53)
Insufficiently active	16 (64)	177 (45.9)		193 (47)
Dependence (Katz Scale)				
Yes	2 (8)	28 (7.3)	0.891	30 (7.3)
No	23 (92)	358 (92.7)		381 (92.7)
Dependence (Lawton and Brody Scale)				
Yes	20 (80)	266 (68.9)	0.243	286 (69.6)
No	5 (20)	120 (31.1)		125 (30.4)
Body mass index (BMI)	21.69 ± 2.12	28.53 ± 4.81	< 0.001	28.11 ± 4.97
Calf circumference (CC)	29.57 ± 2.64	33.45 ± 3.81	< 0.001	33.22 ± 3.86
Arm circumference (AC)	24.10 ± 2.29	29.51 ± 3.70	< 0.001	29.18 ± 3.85
Waist circumference (WC)	90 (79.75–96)	98 (90–105)	< 0.001	97 (90–105)

Data are reported as n: number of subjects; mean ± standard deviation; median (interquartile range); m: meters; kg: kilogram; MMI: muscle mass index; HGS: handgrip strength; kgf: kilogram force; Chi-square test, Student t-test and MannWhitney U test; *p < 0.05

Table 2 presents the adjusted analysis and indicates an independent and inverse association between sarcopenia and the anthropometric indicators, with the increase of one unit of BMI, AC, CC and WC reducing the probability of the subjects to have sarcopenia by approximately 36, 37, 27 and 7%, respectively.

**Table 2:** Association between sarcopenia and anthropometric indicators among community-dwelling older adults

Variables	Sarcopenia		
Anthropometric indicators	OR	95%CI	p* Value
Body mass index (BMI)			
Unadjusted	0.66	0.57–0.76	< 0.001
Adjusted	0.64	0.53–0.76	< 0.001
Calf circumference (CC)			
Unadjusted	0.73	0.64–0.83	< 0.001
Adjusted	0.73	0.62–0.85	< 0.001
Arm circumference (AC)			
Unadjusted	0.63	0.54–0.73	< 0.001
Adjusted	0.63	0.53–0.76	< 0.001
Waist circumference (WC)			
Unadjusted	0.94	0.91–0.97	< 0.001
Adjusted	0.93	0.90–0.97	< 0.001

OR: Odds Ratio; 95%CI: 95% Confidence interval; * *p* < 0.05; Adjusted for age, sex, schooling, income, health perception, number of diseases and medications, hospitalization and occurrence of falls in the last year, smoking habit, level of physical activity, functional disability for basic and instrumental activities of daily life; Hosmer-Lemeshow test (p > 0.05)

**Fig. 1:**
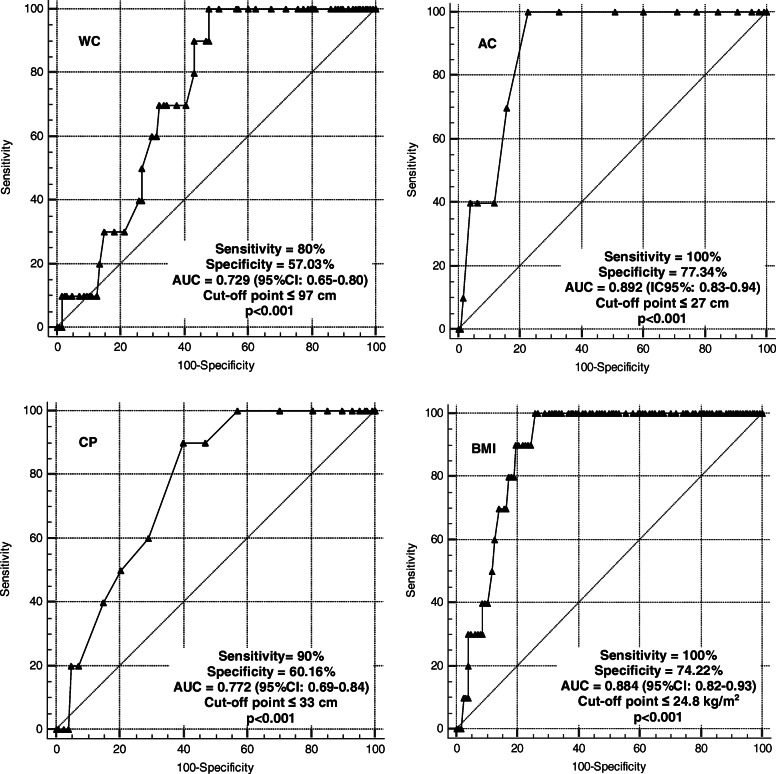
Areas under the ROC curve for the anthropometric indicators as discriminants for the presence of sarcopenia among older men. AUC: area under the ROC curve; CI: confidence interval; WC: waist circumference; AC: arm circumference; CC: calf circumference; BMI: body mass index

**Fig. 2:**
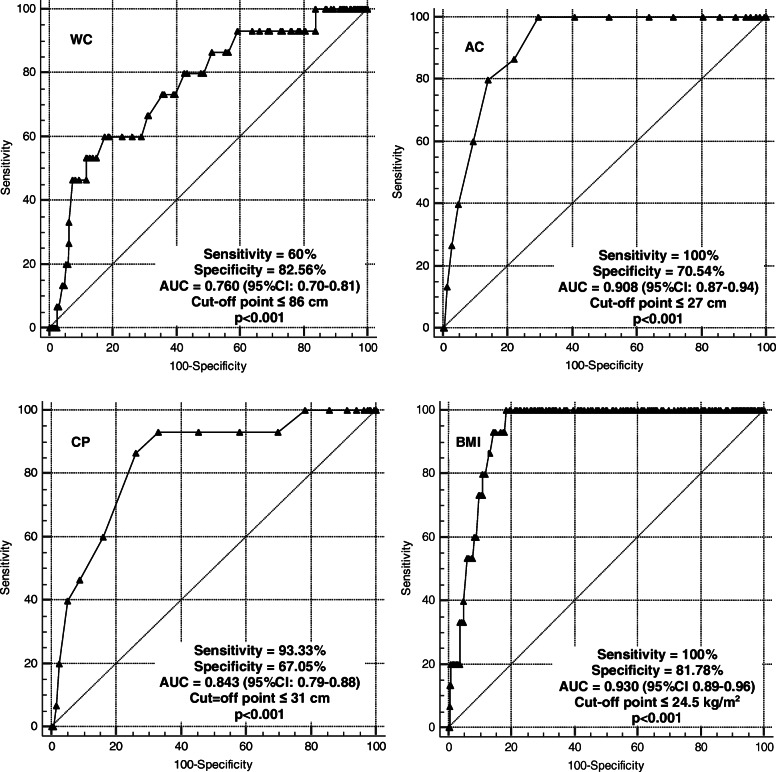
Areas under the ROC curve for the anthropometric indicators as discriminants for the presence of sarcopenia among older women. AUC: area under the ROC curve; CI: confidence interval; WC: waist circumference; AC: arm circumference; CC: calf circumference; BMI: body mass index

The results of the area under the ROC curve indicated coefficients higher than 0.7, representing acceptable discrimination (Figs. 1 and 2). The cut-off points for the older men and women, respectively, represented the discriminant criterion for the presence of sarcopenia, as follows: WC (≤97 and ≤ 86 cm), CC (≤33 and ≤ 31 cm), AC (≤27 cm) and BMI (≤24.8 kg/m^2^ and ≤ 24.5 kg/m^2^) (*p* < 0.05) [38]. BMI and AC were the indicators with the higher ability to discriminate older subjects with sarcopenia of both sexes.

## Discussion

The present study demonstrated an association between all anthropometric indicators and sarcopenia among community-living older adults, with good predictive and discriminatory power [39]. However, the cut-off points that provided the best balance between sensitivity and specificity were ≤ 24.8 kg/m^2^ and ≤ 24.5 kg/m^2^ for BMI among men and women, respectively, and ≤ 27 cm AC for both sexes.

Several studies have shown that anthropometric measurements are useful for the screening of sarcopenia associated with conditions of reduced muscle mass, falls, functionality, and mortality [8, 10, 12, 26, 40, 41]. However, few studies have investigated and validated in a single population all the measurements proposed in the present study.

Regarding CC, the literature has reported a cut-off point of < 31 cm for the screening of sarcopenia [15, 19, 33, 42–44]. A study on the Korean population [8] (mean age: 76.2 years) also reported CC values differing from those reported here, i.e., < 35 for men and < 33 cm for women. In the Japanese population [9] (mean age: 61 years for women and 63 years for men), it were obtained cut-off points for sarcopenia of < 34 cm for men and < 33 cm for women, those exact values were also obtained in a Brazilian study [14] (60 years of age for women and 70 years of age for men). Since the above studies were conducted on an older population of different age ranges, it is difficult to compare their results with the present ones, which involved subjects with a mean age of 77 years. This is confirmed by other studies [10] which reported different and significant results between anthropometric measurements according to age ranges of 60–64, 65–69, 70–74, 75–79, and > 80 years.

Another indicator that showed correlation with sarcopenia in the present study was WC, with cut-off points of ≤97 cm for men (sensitivity = 80% specificity = 57.03%) and ≤ 86 cm for women (sensitivity: 60%, specificity: 82,56%). These results agree with previous study [7] with cut-off points of 92 cm for men (sensitivity: 79.5%, specificity: 66.7%) and 88 cm for women (sensitivity: 65.1%, specificity 85.7%). Also WC, in addition to being a good indicator of sarcopenia, can also be used for the assessment of body composition and central obesity [7].

AC as a single indicator showed association with sarcopenia, with a significant difference from the non-sarcopenic group. The cut-off points obtained were ≤ 27 cm for both sexes, with 100% sensitivity and 77.34% specificity for men and de 100% sensitivity and 70.54% for women. Other studies [10, 25] used corrected arm muscle area and obtained results different from the present ones, with cut-off points of 24.7 cm, 23.8 cm and 21 cm for men and 23.3 cm, 24.7 cm, 23.9 cm e 19.8 cm for women. However, this difference may be justified by the fact that the researchers assessed older subjects aged on average 71.2 years [10] and subjects older than 80 years [25], i.e., older populations than the sarcopenic population investigated here. Although, AC is a valid measure [45] since these authors observed that corrected muscle area is strongly correlated with DEXA for lean mass data. In addition, the AC is the region least susceptible to changes in circumference caused by fluid retention, such as the edema that occurs in the lower limbs [10].

According to data from the present study, the BMI proved to be an acceptable anthropometric instrument for screening sarcopenia for both sexes, with good sensitivity and specificity. This agrees with another study [7] who reported similar cut-off points for BMI with our data [≤24.6 for men (sensitivity: 84.9%, specificity: 63.3%) and ≤ 26.2 for women (sensitivity: 74.6%, specificity: 85.7%)]. Besides, the BMI and advanced age are strongly associated with low musculoskeletal index (appendicular skeletal muscle mass/height) [12].

Anthropometric indicators are easily applied, representing useful measures recommended as part of a screening process since they can be easily obtained at primary health care centers [5, 15, 32, 46–48]. Thus, taking together the information from the present study with the updated recommendation from EWGSOP2 [19], it is proposed for screening sarcopenia an easy pathway to identify people with sarcopenia or its risk, and following this perspective, our data have shown that anthropometric measures could also be an additional and relevant strategy to detected sarcopenia indicators in remote areas such as the Amazon region of the country, mainly in view of the tendency to population aging in Brazil [49].

This data also indicates [49] the existence of a significant population contingent that does not reach the level of consumption of 1900 kcal per day and that is characterized by the consumption of foods with high fat, sugar and salt concentrations, poor variety, and a low consumption of fruits and vegetables. Likewise, reduction in protein intake has been related to the reduction of muscle mass, with a lean mass reduction of as much as 40% within 3 years without the ideal protein intake, which should be about 1–1.2 g/kg [50].

Moreover, it have been reported that several factors may interfere with the prevalence of older adults with sarcopenia such as age, sex, nutrition, geographical region, in addition to individual factors such as percentage of muscle mass, muscle strength and functional capacity [51]. Such factors may explain the divergence of the results obtained here both in relation to studies conducted in developed countries and studies conducted in other regions of Brazil. In Brazil, by the way, the prevalence of sarcopenia was 15.4% in São Paulo-SP [51], 10.8 to 18% among older subjects from Rio de Janeiro-RJ [52], 15.9% in from Pelotas-RS [13] and 17.8% in Lafaiete Coutinho-BA [15]. This clearly shows that divergence exists regarding the characteristics of the population among regions even within the same country.

Some limitations of the study should be considered. The use of the total muscle mass equation [22] offers an estimated calculation; however, it is a method of easy application that does not require expensive equipment and that has been validated and extensively used. Also, by being a cross-sectional study, the present investigation did not permit to infer causality relations between the variables studied. On the other hand, the study provides information about a representative sample of community-living older adults from a municipality of the Amazon region.

In Brazil, the older population increases considerably each year. The vast territory of the country poses many challenges, one of them being, among other aspects, the understanding of how the population aging is occurring in each region. It should be pointed out that a parcel of the population residing in the northern region lives in areas of difficult access for both older people and health agents, aspects that can impair the implementation of actions. Thus, it is clearly important to identify new tools of easy access and handling for the early screening of sarcopenia, especially among older adults living in the northern region of Brazil. According to the results of the present study, anthropometric measures proved to be effective for this purpose, thus permitting the development of new preventive and therapeutic strategies for this population.

## Conclusion

Sarcopenic older adults had lower mean values of anthropometric measurements than non-sarcopenic subjects. An increase of one unit of these indicators may reduce the probability of the occurrence of sarcopenia among community-living older adults. All anthropometric indicators were considered to discriminate for sarcopenia with the cut-off points for BMI and AC showing a better equilibrium in the sensitivity and specificity relationship.

## Supplementary Information

**Additional file 1.**

## Data Availability

The datasets used and/or analysed during the current study are available from the corresponding author on reasonable request.

## Abbreviations

AC: Arm circumference
WC: Waist circumference
CC: Calf circumference
BMI: Body mass index
OR: Odds ratio
CI: confidence intervals
DXA: Dual-energy X-ray emission absorptiometry
MR: Magnetic resonance
CT: Computed tomography
WHO: World Health Organization
AbC: Abdominal circumference
MMSE: Mini Mental State Examination
EWGSOP 2: European Working Group on Sarcopenia in Older People
TMM: Total muscle mass
MMI: Muscle mass index
HGS: Handgrip strength
BADL: Basic activities of daily life
IADL: Instrumental activities of daily life
IPAQ: International Physical Activity Questionnaire
ROC: Receiver Operating Characteristic
SPSS: Statistical Package for the Social Sciences
AUC: Area under the ROC curve
