# Supplementary material for: Anthropometric indicators as a discriminator of sarcopenia in community-dwelling older adults of the Amazon region: a cross-sectional study
Source: BMC Geriatr. 2020 Dec 1;20:518. doi: 10.1186/s12877-020-01923-y (PMC7709449; doi:10.1186/s12877-020-01923-y)
Supplement: Supplementary file 1 — Additional file 1. [file 12877_2020_1923_MOESM1_ESM.docx]

**IDENTIFICATION**

**Name:**_______________________________________________________ **Code:**

Interview time: Start __________ End ___________ Date of Collection: ____ / ____ / ______

District:______________ Address:________________________________________Ph: __________

Sensus Sector: ___________________________

**1) Date of Birth:** ____/____/______ (Day/Month/Year)

Age: (full years)**......................................................................................................................**

ATTENTION:

- The interviewee must be 60 years or older. Ask the interviewee for an identification document showing the date of birth.
- If the person **does not** meet the above criteria, thank them and finished the interview.

**2) Sex...............................................................................................................................................**

1- Male 2- Female

**3) Schooling:......................................................................................................................................**

Attention: it should be noted how many years studied without repeating the same grade.

**MINI-MENTAL STATUS EXAMINATION (MMSE)**

**4) Orientation to Time** (1 point for each correct answer or 0 if not responding adequately)

Em que ano estamos? _____

What is the year? _____

What is the month? _____

What is today's date? _____

What is the day of the week today? _____

What season is it? ­

**Score:____**

**5) Orientation to Place** (1 point for each correct answer or 0 if not responding adequately)

What state are we in? _____

What city/town are we in? _____

What is the street address we in? _____

What place are we in? _____

What specific place are we in (point to the ground)? _____

**Nota:____**

**6) Immediate Recall** (Put 1 point for each correctly repeated word or 0 when the elder does not repeat the word correctly)

"I am going to name three objects. When I am finished, I want you to repeat them. Remember what they are because I am going to ask you to name them again in a few minutes” .

Mug _____

Carpet _____

Brick _____

**Score: ____**

**7) Attention and Calculation** (In the spaces below add 1 if the answer is correct and 0 for wrong answer. In the "score” put the sum of the correct answers).

a) "Now, I ask you to tell me the result of 100 minus 7 and then the number found again to take 7 and repeat so until I tell you to stop".

100______ 93_____ 86_______ 79_______ 72______ 65

**Score:_______**

b) “Spell the word WORLD backwards”

D____ L____ R____ O_____ W_____

**Score:** _______

*Consider in the Final Score of question 8 the highest Score between items a and b*.

**Final Score:** _________

**8) Delayed Verbal Recall** (1 point for each correct answer or 0 when not correct)

“Now what were the three objects I asked you to remember?”

Mug _____

Carpet _____

Brick _____

**Score:____**

**9) Language** (1 point for each correct answer or 0 when not correct)

a. "What is this called?” Show the following objects:

Watch ____

Pencil ______

**Score:____**

b. "I would like you to repeat a phrase after me: No ifs, ands, or buts”

**Score:____**

c. "“Take the paper in your right hand, fold it in half, and put it on the table”; (The examiner gives the patient a piece of blank paper with both hands)

Takes ____

Folds ____

Puts ____

**Score:____**

d. "Read the words on this page and then do what it says” . Hold up the card reading: "Close your eyes" so the individual can see it clearly, or or read the card in case the elderly person is illiterate.

**Score:____**

e. "Write any complete sentence on that piece of paper”. It must contain a subject and verb and be sensible; Ignore spelling erros.

Setence:

**Score:____**

**10) Visual Constructive Capacity** (1 point for correct copy)

It must to draw the symbol below. Two partially overlapping pentagons; each one should have 5 sides, two of which intersect. Do not value tremor or rotation.


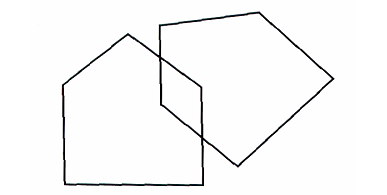


**Score:____**

**11) TOTAL SCORE (maximum 30 points): _______**

**SOCIOECONOMIC VARIABLES**

**12) What race do you consider?.....................................................................................................**

1-~~White~~ Caucasian 2-Black 3-Brown 4-Asian 5-Native

**13) What is your marital status? ......................................................................................................**

1- Never married or never lived with a partner

2- Lives with husband/wife or partner

3- Widower/widow

4 –Separated or divorced 99- Ignored

**14) What’s your individual income?......................................................................................................**

1 - No income 2 - Less than 1 minimum wage 3 - 1 minimum wage 4 – 1-3 minimum wages 5 – 3-5 minimum wages 6 - more than 5 minimum wages

**15) Its financial resources currently come from: (Multiple choice).....................**

1-Retirement 2-Pension

3-Income/rent 4-Donation (family)

5-Donation (other) 6-Continuous work (formal or not)

7- Occasional work 8-Monthly income for life

9- Financial application 10- No own income

99- Ignored

**16) Main professional activity currently exercised:....................................................................**

1- Housewife 2- Housekeeper 3- Manual labour

4- Rural worker 5- Professional person 6- Farmer 7- Businessman

8- Other :_______________________) 9- Does not perform 99- Ignored

**17) What’s the reason you retired?..............................................................................................**

1- Service time 2- Age 3- Health Issue: ________________________

4- It is not retired 99- Ignored

**18) According to your understanding according to your economic situation, how do you meet your basic needs? (food, housing, health, etc.) ......................................................................**

1- Bad 2- Regular 3- Good 99- Ignored

**19) In general and in comparison to the economic situation of other people your age, you would say that your economic situation is...? .........................................................................**

1- Worse 2- Egual 3- Better 99-Ignored

**20) The house where you live is:..................................................................................................**

1- own home - paying off 2- own home - mortgage 3- relatives – pay rent

4- diferente person – pay rent 5- house provided/house given – no rent 6- asylum 99- Ignored

**21) You live in your home.... (Multiple choice):.................................................................**

(1) Alone (no one else lives permanently together)

(2) With professional caregiver (1 or +)

(3) With the spouse

(4) With others of the same generation

(5) With children

(6) With grandchildren

(7) With daughter-in-law or son-in-law

(8) Others _________________________________

(9) Does not Know (99) Did not answer

**PHYSICAL HEALTH**

**22) Overall, how would you say your health is? ....................................................................**

1- Terrible 2- Bad 3- Regular 4- Good 5- Great 99- Ignored

**23) Comparing your health today to that of 12 months ago, how is your health? .................**

1- Worse 2- Egual 3- Better 99- Ignored

**24) Compared to the health of other people your age, the (a) lord(a) would say that your health is:.........................................................................................................................................**

1- Worse 2- Egual 3- Better 99- Ignored

**25) Do you currently have any of these health problems?**

| **Morbidity** | **Yes** | **No** | **Ignored** |
| --- | --- | --- | --- |
| A)Rheumatism | 1 | 2 | 99 |
| B)Arthritis/arthritis | 1 | 2 | 99 |
| C)Osteoporosis | 1 | 2 | 99 |
| D)Asthma or bronchitis. | 1 | 2 | 99 |
| E)Tuberculosis | 1 | 2 | 99 |
| F)Embolism | 1 | 2 | 99 |
| G)High blood pressure | 1 | 2 | 99 |
| H)Poor circulation (varicose veins) | 1 | 2 | 99 |
| I)Heart problem | 1 | 2 | 99 |
| J)Diabetes | 1 | 2 | 99 |
| K)Obesity | 1 | 2 | 99 |
| L)Stroke | 1 | 2 | 99 |
| M)Parkinson’s disease | 1 | 2 | 99 |
| N)Urinary Incontinence | 1 | 2 | 99 |
| O)Faecal Incontinence | 1 | 2 | 99 |
| P)Constipation | 1 | 2 | 99 |
| Q)Trouble sleeping | 1 | 2 | 99 |
| R)Cataract | 1 | 2 | 99 |
| S)Glaucoma | 1 | 2 | 99 |
| T)Back problems | 1 | 2 | 99 |
| U)Renal issues | 1 | 2 | 99 |
| V)Accident / trauma sequel | 1 | 2 | 99 |
| W)Malignant tumors | 1 | 2 | 99 |
| Y)Benign tumors | 1 | 2 | 99 |
| Z)Problem of vision | 1 | 2 | 99 |
| Depression | 1 | 2 | 99 |
| Another____________________________ | 1 | 2 | 99 |
|  | 1 | 2 | 99 |
|  | 1 | 2 | 99 |
|  | 1 | 2 | 99 |
|  | 1 | 2 | 99 |
|  | 1 | 2 | 99 |

**26) Number of Diseases ......................................................................................................................**

**27) Write down the number of medications regularly used by the elderly and describe them below .........................................................................................................................................**

| **Name (active principle)** | **Dosage** | **(1) µg (2) mg (3) g**  **(4) UI (5) mg/ml**  **(6) Others (Which one?)** | **Amount per day** |
| --- | --- | --- | --- |
|  |  |  |  |
|  |  |  |  |
|  |  |  |  |
|  |  |  |  |
|  |  |  |  |
|  |  |  |  |
|  |  |  |  |
|  |  |  |  |
|  |  |  |  |
|  |  |  |  |
|  |  |  |  |
|  |  |  |  |

**Do you currently smoke any tobacco products?**

1. Yes, daily 2. Sim, less than daily 3. I do not smoke currently

**And in the past, have you smoked any tobacco products daily?**

1. Yes 2. No

**And in the past, have you smoked any tobacco products?**

1. Yes, daily 2. Yes, less than daily 3. No, I never smoked

**How old were you when you started smoking cigarettes every day?** ________ years

**How often do you drink alcohol?**

1. I never drink 2. Less than once a month 3. Once or more a month

**How many days a week do you usually drink alcohol?**

Days ( ) Never or less than once a week ( )

**In general, on the day you drink, how many doses of alcohol do you consume? (1 dose of alcoholic beverage equals 1 can of beer, 1 glass of wine or 1 dose of cachaça, whiskey or any other distilled alcoholic drink** ( ) shots

**In the last 30 days, have you consumed 5 or more doses of alcohol on a single occasion? (If it is a man)** (1) or

**In the last 30 days, have you consumed 4 or more doses of alcohol on a single occasion? (If it is a woman)** (2)

1) yes 2) No

**How many days in the month did this occur?**

1. 1 day 2) 2 days 3) 3 days 4) 4 days 5) 5 days 6) 6 days 7) 7 or more

**1) Have you been hospitalized in the last twelve months (last year)?.......................................**

1) yes 2) no

**2) How many times? .........................................................................................................................**

**1) Did you fall in the last 12 months? ........................................................................................**

(0) NO (1) YES

If the answer is NO, go to FALLS EFFICACY SCALE

**2) How many times have you fallen in the last 12 months? ........................................................**

(99) NS/NR

**MENTAL HEALTH**

**GERIATRIC DEPRESSION SCALE (GDS -15)**

**For each question below answer (circle) "No" or "Yes". In "Total" put the final score obtained**

|  |  | **Não** | **Sim** |
| --- | --- | --- | --- |
| **17** | Are you basically satisfied with your life? | **1** | 0 |
| **18** | Have you dropped many pof your activities and interests? | 0 | **1** |
| **19** | Do you feel that your life is empty? | 0 | **1** |
| **20** | Do you often get bored? | 0 | **1** |
| **21** | Are you in good spirits most of the time? | **1** | 0 |
| **22** | Are you afraid something bad is going to happen to you? | 0 | **1** |
| **23** | Do you feel happy most of the time? | **1** | 0 |
| **24** | Do you often feel helpless? | 0 | **1** |
| **25** | Do you prefer to stay at home, rather tha going out and doing new things? | 0 | **1** |
| **26** | Do you feel you have more problems with memory than most? | 0 | **1** |
| **27** | Do you think it is wonderful to be alive? | **1** | 0 |
| **28** | Do you feel pretty worthless the way you are now? | 0 | **1** |
| **29** | Do you feel full of energy? | **1** | 0 |
| **30** | Do you feel your situation is hopeless? | 0 | **1** |
| **31** | Do you think that most people are better off than you are? | 0 | **1** |
| **TOTAL** | |  | |

Over 5 indicates depression.

**Presence of Indicative of depression:.............................................................................**

(1) Yes (2) No

**FUNCTIONAL CAPACITY**

**KATZ INDEX OF INDEPENDENCE OF DAILY LIVING**

| **Activity** | **Indep.**  **Dep.** |
| --- | --- |
| **Bathing** |  |
| 1. No supervision, direction or personal assistence | (1) |
| 2. Receive help to wash only a part of the body (such as the back or a leg). | (2) |
| 3. Need help with bathing more than one part of the body, getting in or out of the tub or shower. Requires total bathing | (3) |
| **Dressing** |  |
| 1. Take the clothes and dress completely, without help | (1) |
| 2. You take the clothes and dress without help, except to tie the shoes | (2) |
| 3. Get help to pick up clothes or dress up, or stay partially or completely without clothes | (3) |
| **Going to Toilet** |  |
| 1. Go to the bathroom or equivalent place, clean up and fix the clothes without help (can use objects for support as cane, walker or wheelchair and can use bedpan or urinal at night, emptying it in the morning) | (1) |
| 2. Get help to go to the bathroom or equivalent place, or to clean yourself, or to clean clothes after evacuation or urination, or to use bedpan or urinal at night | (2) |
| 3. Does not go to the bathroom or equivalent for physiological elimination | (3) |
| **Transferring** |  |
| 1. Lies down and gets out of bed, sits down and gets up from the chair unaided (may be using object for support, such as walking stick or walker) | (1) |
| 2. Lie down and get out of bed and/or sit down and get up from the chair with help | (2) |
| 3. doesn't get out of bed | (3) |
| **Continence** |  |
| 1. Fully controls urination and evacuation | (1) |
| 2. Has occasional "accidents" | (2) |
| 3. Needs help to maintain urination control and evacuation; uses catheter or is incontinent | (3) |
| **Feeding** |  |
| 1. Feeds without assistance | (1) |
| 2. Eats alone, but receives help to cut meat or butter bread | (2) |
| 3. Receives food aid or is fed partially or completely by the use of catheters or intravenous fluids | (3) |

0: independent in all six functions;

1: independent in five roles and dependent in one function;

2: independent in four roles and dependent in two;

3: independent in three roles and dependent in three;

4: independent in two roles and dependent in four;

5: independent in one function and dependent in five functions;

6: dependent in all six funçctions

**Classification.......................................................................................................................................**

**Number of ADL you cannot perform ................................................................................................**

**INSTRUMENTAL ACTIVITIES OF DAILY LIVING SCALE (I.A.D.L.) - LAWTON E BRODY SCALE**

| **Activities** |  | |
| --- | --- | --- |
| Ability to Use Telephone | | |
| a) Telephone | Operates telephone on own initiative  Needs assistance to make phone calls  Not in the habit or unable to use the phone | 3  2  1 |
| Mode of Transportation. | | |
| b) Travel | Travels alone  Only when it has company  Not in the habit or unable to travel | 3  2  1 |
| Going to shop | | |
| c) Shoping | Makes purchases when transport is provided.  You only shop when you have company.  It is not in the habit or unable to make purchases. | 3  2  1 |
| Food preparation | | |
| d) Food | Plan and cook the complete meals.  Only prepares small meals or when gets help.  It has no habit or is unable to prepare own meals. | 3  2  1 |
| Housekeeping | | |
| e) Housekeeping | It performs heavy tasks.  It performs light tasks, needing help with the heavy ones.  It is not in the habit or unable to do housework. | 3  2  1 |
| Responsibility for Own Medications | | |
| f) Medications | Makes use of medicines without assistance. Need reminders or assistance.  Unable to control alone the use of medicines | 3  2  1 |
| Ability to Handle Finances | | |
| g) Finaces | It writes checks and pays unassisted bills.  Need assistance with checks and bills.  Not in the habit of dealing with money or unable to handle money, bills... | 3  2  1 |

**TOTAL..................................................................................................................................................**

**Classification of dependency for IADL** **.............................................................................................**

(1) Total dependency: equal to 7 points

(2) Partial dependency: 7 21 points

(3) Independent: 21 points

1. **Unintentional weight loss the last year**

| In the past year, have you lost more than 4.5 kg unintentionally (i.e., without diet or exercise)? (1) yes (2) no |
| --- |
| If so, how many kilograms approximately? kg |

1. **Muscle Strength / decrease of the dominant hand grip force measured by the dynamometer and adjusted to sex and body mass index**

| 1st measure of grip force |  | 2nd measure of grip force |  | 3rd measure of grip force |  |
| --- | --- | --- | --- | --- | --- |
| **Average:________** | | | | | |

1. **Self Reporting of Exhaustion/Fatigue**

Thinking about the last week, say how often the following things have happened to you

| QUESTION | | NEVER/RARELY | NOT OFTEN | MOST OFTEN | USUAL |
| --- | --- | --- | --- | --- | --- |
| Did you feel you had to make an effort to accomplish your usual tasks? | (1) Yes  (2) No | (1) | (2) | (3) | (4) |
| Have you been unable to carry on with your things? | (1) Yes  (2) No | (1) | (2) | (3) | (4) |

1. **Slowness/gait speed**

Mr./Mrs. Do you usually use an auxiliary gait device, such as a walking stick or walker? (1) does not use (2) Walker (3) Cane (4) Other

| 1st measure gait speed |  | 2nd measure gait speed |  | 3rd measure |  |
| --- | --- | --- | --- | --- | --- |
| **Average:________** | | | | | |

1. **Physical Activity / Low physical Activity**

To answer the questions remember that:

- VIGOROUS physical activities are those that require great physical effort and that make breathing MUCH stronger than normal;
- MODERATE physical activities are those that require some physical effort and that make breathing A little stronger than normal;
- LIGHT physical activities are those in which physical exertion is normal, making breathing normal.

***Score (section 1+ section2 + section3 + section4) = __________min/sem***

**SECTION 1- Occupational Physical Activity** *Time (1b + 1c +1d) = ______min*/*sem*

In this section are the activities you do in your service, which include paid or voluntary work, school or college activities (intellectual work) and other unpaid work outside your home, DO NOT include the tasks you do in your home, such as household chores, gardening and housekeeping, or taking care of your family. These will be included in section 3.

**1a. Do you currently work or do you do volunteer work?**

(1) Yes (2) No ( If you answer "no" ***Go to section 2: Transport)***

The next issues are related to all the physical activity you do in a normal or usual week as part of your paid or unpaid work, not including transportation to work. Think only of the activities you do for at least 10 continuous minutes:

**1b.** On how many days of a normal week do you spend doing vigorous activities, for at least 10 continuous min, such as heavy construction work, carrying large weights, working with hoe, chopping wood, sawing wood, mowing grass, painting house, digging ditches or holes, Climbing stairs as part of your job? (Place the number of minutes on each day of the week in the table below and the total number of minutes below)

TOTAL: _________minutes, ( ) none – **Go to question 1c**

| Day | Mondey | Tuesday | Wednesday | Thursday | Friday | Saturday | Monday |
| --- | --- | --- | --- | --- | --- | --- | --- |
| Time minutes |  |  |  |  |  |  |  |

**1c.** On how many days of a regular week do you do moderate activities, for at least 10 continuous minutes, such as carrying light weights, cleaning glass, sweeping or cleaning the floor, carrying children on your lap, washing clothes with your hand as part of your paid or voluntary work?

________minutes ( ) none – **Go to question 1d**

| Day | Mondey | Tuesday | Wednesday | Thursday | Friday | Saturday | Monday |
| --- | --- | --- | --- | --- | --- | --- | --- |
| Time minutes |  |  |  |  |  |  |  |

**1d.** On how many days of a regular week do you walk/walk for at least 10 continuous minutes as part of your job? Please DO NOT include walking as a means of transportation to go to or back from work or the place you volunteer

________minutes ( ) none – **Go to section 2 – Transport**

| Day | Mondey | Tuesday | Wednesday | Thursday | Friday | Saturday | Monday |
| --- | --- | --- | --- | --- | --- | --- | --- |
| Time minutes |  |  |  |  |  |  |  |

***SECTION 2 - Physical Activity as a Means of Transport***

*Time (2a +2b +2c) =: ______min*/*sem*

. These issues refer to the normal way you move from one place to another, including your work, school, fair, church, cinema, shops, supermarket, senior group meeting or anywhere else.

**2a.** How many days in a normal week do you drive, bus or motorcycle?

________minutes ( ) none – **Go to question 2b**

| Day | Mondey | Tuesday | Wednesday | Thursday | Friday | Saturday | Monday |
| --- | --- | --- | --- | --- | --- | --- | --- |
| Time minutes |  |  |  |  |  |  |  |

**Now think only of walking or cycling to go from one place to another in a normal week.**

**2b.** On how many days of a regular week do you ride your bike for at least 10 continuous minutes to go from one place to another? (NOT including cycling for leisure or exercise).

_______ minutes ( ) None – **Go to question 2c**

| Day | Mondey | Tuesday | Wednesday | Thursday | Friday | Saturday | Monday |
| --- | --- | --- | --- | --- | --- | --- | --- |
| Time minutes |  |  |  |  |  |  |  |

**2c.** On how many days of a normal week do you walk for at least 10 continuous minutes to go from one place to another, such as: going to the living group for the elderly, church, supermarket, fair, doctor, bank, visit a relative or neighbor? (DO NOT INCLUDE hiking for pleasure or exercise).

______ minutes ( ) None – **Go to section 3**

| Day | Mondey | Tuesday | Wednesday | Thursday | Friday | Saturday | Monday |
| --- | --- | --- | --- | --- | --- | --- | --- |
| Time minutes |  |  |  |  |  |  |  |

***SECTION 3 - Physical activity at home: work, housework and family care***

*Time (3a + 3b + 3c)= ______min*/*sem*

This part includes the physical activities you do in a normal/usual week in and around your home, for example, work at home, take care of the garden, take care of the yard, work maintenance of the house or to take care of your family. Again, just think about those physical activities you do for at least 10 continuous minutes.

**3a**. On how many days of a normal week do you do vigorous physical activities in the garden or yard for at least 10min such as: carping, washing the yard, scrubbing the floor, chopping wood, painting house, lifting and carrying heavy objects, mowing grass with scissors:

_______ minutes ( ) none – **Go to question 3b**

| Day | Mondey | Tuesday | Wednesday | Thursday | Friday | Saturday | Monday |
| --- | --- | --- | --- | --- | --- | --- | --- |
| Time minutes |  |  |  |  |  |  |  |

**3b.** On how many days of a normal week do you do moderate activities in the garden or yard for at least 10 min such as: carrying light weights, cleaning windows, sweeping, cleaning the garage, playing with children, raking the grass, gardening service in general.

________ minutes ( ) None – **Go to question 3c.**

| Day | Mondey | Tuesday | Wednesday | Thursday | Friday | Saturday | Monday |
| --- | --- | --- | --- | --- | --- | --- | --- |
| Time minutes |  |  |  |  |  |  |  |

**3c.** On how many days of a normal week you do moderate activities inside your home for at least 10 minutes like carrying light weights, cleaning windows or windows, washing clothes by hand, cleaning toilets, sweeping or cleaning the floor.

_______ minutes ( ) None – **Go to section 4**

| Day | Mondey | Tuesday | Wednesday | Thursday | Friday | Saturday | Monday |
| --- | --- | --- | --- | --- | --- | --- | --- |
| Time minutes |  |  |  |  |  |  |  |

**SECTION 4, Recreation, Sport, Exercise and Leisure Physical Activities**

***Time (4a + 4b + 4c) = ___________min/sem***

This section refers to the physical activities you do in a normal week solely for recreation, sport, exercise or leisure. Again think only of the physical activities you do for at least 10 continuous minutes. Please DO NOT include activities you have already mentioned.

**4a**,Not counting any hike you take as a means of transportation (to move from one place to another), on how many days of a normal week, do you walk for at least 10 continuous minutes in your free time?

_______ minutes ( ) None – **Go to question 4b**

| Day | Mondey | Tuesday | Wednesday | Thursday | Friday | Saturday | Monday |
| --- | --- | --- | --- | --- | --- | --- | --- |
| Time minutes |  |  |  |  |  |  |  |

**4b**. On how many days of a normal week, you do vigorous activities in your free time for, me/us, 10 min, such as running, fast swimming, weight training, rowing, fast cycling, in short sports in general:

_______ minutes ( ) None – **Go to question 4c**

| Day | Mondey | Tuesday | Wednesday | Thursday | Friday | Saturday | Monday |
| --- | --- | --- | --- | --- | --- | --- | --- |
| Time minutes |  |  |  |  |  |  |  |

**4c**. On how many days of a normal week, you do moderate activities in your free time for at least 10 min, such as cycling or swimming at regular speed, playing ball, volleyball, basketball, tennis, swimming, water aerobics, gymnastics for seniors, dancing and petting.

_______ minutes ( ) None – **Go to question 5**

| Day | Mondey | Tuesday | Wednesday | Thursday | Friday | Saturday | Monday |
| --- | --- | --- | --- | --- | --- | --- | --- |
| Time minutes |  |  |  |  |  |  |  |

**SECTION 5 - Time Spent Sitting**

These last questions are about the time you sit in different places, such as at work, at school or college, at home, in the living group for the elderly, in the doctor’s office and during your free time, This includes sitting time while resting, watching TV, doing manual work, visiting friends and relatives, take readings, phone calls, at Mass/worship and hold meals. Do not include time spent sitting during transportation by bus, car or motorcycle.

**5a.** How much time in total do you spend sitting on a weekday?

_________hours _______minutes

**5b.** How much time in total do you spend sitting on a weekend day?

_________hours _______minutes

**ANTHOPOMETRIC DATA**

*(Write down the answers in the next spaces)*

**30) Weight (Kg)........................................................................................................................**

**31) Height (m): ..............................................................................................................................**

**32) Abdominal Circumference (cm)................................................................................................**

**33) Calf Circunference (cm):.........................................................................................**

**34) Brachial Circumference (cm):..................................................................................................**
